# Robots for learning: an exploration of teacher roles, perceptions, and challenges in robot-mediated learning

**DOI:** 10.3389/frobt.2025.1441382

**Published:** 2025-07-01

**Authors:** Veronica Ahumada-Newhart, Jacquelynne S. Eccles

**Affiliations:** ^1^ School of Medicine, University of California Davis, Sacramento, CA, United States; ^2^ School of Education, University of California Irvine, Irvine, CA, United States

**Keywords:** robots, teaching, learning, inclusion, telepresence, robot design, user experience

## Abstract

With the increasing popularity of robots for learning, many educational organizations are using telepresence robots for the purpose of remote education. However, as robot-mediated learning is important for the learning experiences of remote and local interactants, it is also important to understand teacher roles and robot design features needed to facilitate these roles in robot-mediated learning experiences. In this paper, we present findings from analysis of a national, multi-case study, where we explore how (*N* = 60) K-12 teachers perceive their roles in teaching hybrid classrooms where a remote learner used a robot to attend a physical classroom with in-person classmates. This paper presents a qualitative study of the perceived roles for teachers in hybrid classrooms where a remote learner uses a telepresence robot to participate in learning activities. In 46 semi-structured interviews (*n =* 46) and 6 focus group interviews (*n* = 2; *n* = 3; *n* = 2; *n* = 3; *n* = 2; *n* = 2), coded with a computer assisted qualitative data analysis software (i.e., ATLAS.ti), we captured adapted roles enacted by teachers in robot-mediated learning experiences. First, we present empirical data on educator perceptions of teacher roles as they interact with mobile telepresence robots embodied by remote learners. Specifically, we explore perceptions and roles during in-class learning activities, in-school social activities, and learning preparation activities. Findings from our work will inform novel teacher-centered robot and HRI design that facilitates teaching hybrid classrooms. Findings will also inform future interdisciplinary studies on robot-mediated learning.

## 1 Introduction

With the increasing popularity of robots for learning, many educational organizations are using telepresence robots for the purpose of remote education. Earlier studies have focused on the efficacy of robots for remote and local learners but very few studies have focused on teacher roles and robot design features needed to facilitate these roles in robot-mediated learning experiences. Teachers facilitate learning using multiple dynamic approaches and techniques. In addition to lecture-based instruction, teachers employ one-to-one (i.e., teacher-to-student), peer pairs, and groupwork settings that may create teaching challenges when a learner is present via robot. Current teacher roles are established for in-person learning and require some modifications for teaching classrooms where a physical robot is moving about and interacting in the physical space. In this scenario, the robot’s body, mobility, audio, and video capabilities are synthetic and, at times, restricted due to robot body design, quality of connection, design of physical spaces, and human noise levels. For teachers, additional teaching skills and responsibilities are necessary when including remote robot-mediated learners in their lessons.

The importance of a teacher’s role in the learning process is widely recognized (Franklin and Harrington, 2019) and consists of many culturally defined roles (Pettersson et al., 2004). Traditionally, teacher roles have included class leader, lecturer, information giver, and discussion leader (Murchú, 2005). As technology has evolved into classroom settings, new roles have emerged as instructional designer, trainer, collaborator, team coordinator, advisor, and monitoring and assessment specialist (Murchú, 2005). However, these roles emerged as significant for teachers who led classrooms where learners were all in-person experiencing novel technologies together. Both traditional and technology-centered teacher roles have typically been associated with specific activities, or “tasks” made possible by the use of existing resources in support of individual, group, and/or project-based learning.

**FIGURE 1:**
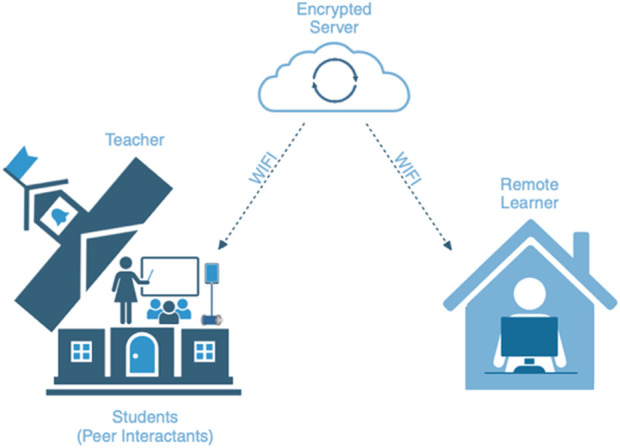
Robot-mediated learning.

For this study, our guiding research question was, “What are the perceptions of teacher roles in the robot-mediated learning experience?” All participants in our study were teachers who led their classrooms in experiencing a novel technology (i.e., telepresence robot) through multiple platforms. The teacher and classmates experienced in-person learning with a mobile robot body in the classroom and the remote learner experienced online virtual learning through a laptop/desktop computer. Teacher, classmates, and learners also experienced interacting through the digital audio and digital video capabilities of either the robot or a computer. All robot-mediated teaching and learning activities were mediated through a connectivity path that took place from home WIFI to an encrypted server to the school’s WIFI (Figure 1).

### 1.1 Background

Earlier work has explored telepresence robots used in teacher roles (Kupferman, 2022; Blar et al., 2014; Kanda et al., 2007; Movellan et al., 2009; Wang et al., 2023; Wernbacher et al., 2022; Leoste et al., 2022) and telepresence robots used in learner roles (Ahumada-Newhart and Eccles, 2020; Weibel et al., 2023; Gallon et al., 2019; Ahumada-Newhart and Olson, 2019; Page et al., 2021; Newhart et al., 2016). However, very few, if any, studies have explored the roles of teachers when teaching to a telepresence robotic unit embodied by a remote learner. Our rationale for this study is centered on an urgent need to better understand the role of the teacher in robot-mediated learning for more careful design of future robots and human/robot positioning in learning environments. Such understanding may lead to creation of improved robot bodies, artificial intelligence, audio/video capabilities, and human/robot practices that facilitate teacher roles in robot-mediated learning.

Mobile telepresence robots have capabilities that allow remote learners to interact with their teachers and peers for social and academic learning in traditional physical environments. These robots provide remote learners with opportunities to build on their personal foundational knowledge of social interactions and experiences to create new scenarios of learning and creativity.

In this paper, we report data from a national, multi-case study of teachers in grades K-12 who taught classrooms where remote learners used mobile telepresence robots for learning. The main research question guiding our national, ongoing, study is, “How are mobile telepresence robots used in schools by remote children, their teachers, and classmates?” This paper is focused on the roles of teachers in the classroom.

Out of 60 teacher cases in our national study, 1 case reported refusing to use the robot in the classroom and 59 cases reported participation in at least one of five core teacher roles in robot-mediated learning:

1) Capacity building (preparing instructional materials, securing digital tools, connections, access);
2) Environment design (placement of learners, robot, desks, classroom resources)
3) Instruction
  a. Lecture—lecture-based instruction to the whole class w/robot-mediated communications
  b. Discussion leader—monitor individual and one-on-one robot communications in the classroom
  c. Team leader—groupwork >2 children, groupwork <2
4) Mentorship and guidance
  a. Individual in-class and non-robot external communications
5) Assessment and evaluation (physical and online grading)

Data for this paper were collected from 2016–2022 through semi-structured interviews, focus groups, and field notes. As a subset of a larger study, data for this paper consisted of cases where participants were classroom teachers who taught classrooms where a remote student used a mobile telepresence robot to attend class.

The contributions of this paper are as follows. First, we present empirical data on novel teacher roles that emerged organically through use of telepresence robots in the classroom. These teacher roles emerged in five categories: capacity builder, assessment, instruction, advisor, environment designer. Second, we explore unique challenges that emerged for teachers when telepresence robots were used for learning in the classroom. Third, our work found that perceptions of the robot varied based on perceived utility of the robot and accessibility. Understanding how teachers interact with robotic units in the classroom can inform future design of robots to facilitate whole class, group, paired, and one-on-one teaching scenarios. Additionally, understanding how robots interact within physical learning environments can inform future interdisciplinary HRI studies and built environments.

## 2 Related work

### 2.1 Teacher roles

Teachers fill a complex set of roles, which vary from one society to another and from one educational level to another. On a global level, teachers play a key role in shaping the future, unlocking every learner’s potential, and achieving global goals of inclusive and equitable quality education (UNESCO, 2024). The major role of a teacher is to support learners in their quest for new knowledge on a specified set of subjects (NCEE, 2020). How teachers facilitate new knowledge has fundamentally changed with evolving technologies in the classroom. Teaching and instruction do not consist primarily of lecturing to individuals who sit in rows at desks, dutifully listening and recording what they hear. Ideally, teachers provide every learner with a rich, rewarding, and unique learning experience. Additionally, with increasing use of the internet in schools, teachers are responsible for moderating learning environments that are not confined to the classroom but, instead, extend into the home and the community and possibly around the world. Information is not bound primarily in books; it is available everywhere in bits and bytes (Lanier, 1997). Teachers are continually tasked with assessing and adapting their roles as shifts occur in digital access to knowledge, educational policies, local communities, families, society, and technologies. As such, the introduction of mobile robots in learning environments requires some modification of traditional teacher roles.

### 2.2 Teacher motivation

#### 2.2.1 Why do teachers participate in robot-mediated learning?

Research has identified the highest motivations for teaching were perceived teaching abilities, the intrinsic value of teaching, the desire to make a social contribution, and shape the future. The Eccles et al., Situated Expectancy-Value Theory (SEVT), originally proposed by Eccles and her colleagues (Wigfield and Eccles, 2000; Eccles and Wigfield, 2002; Eccles Parsons, 1983) to study academic achievement among students and adapted for use among teachers by Abrami et al. (2004), is a promising explanatory model for detailing the influences on teachers’ instructional decision making because it encompasses both beliefs and the anticipated costs associated with making particular decisions within particular contexts (Day, 2020). According to SEVT, people are motivated to engage in particular tasks (including teachers in classrooms) to the extent that they have high expectations about success, and the potential value of engaging in the task is greater than the perceived costs of engaging in the activity or teaching strategy in the case of teachers. In this study, we extend Situated Expectancy-Value Theory (SEVT) as a theoretical framework for understanding the complex relationship between teacher’s beliefs and practices in adapting teacher roles for robot-mediated learning. Our proposed EVT framework has the capacity to capture subjective task values and decision-making factors in the robot-mediated context within a single framework (Table 1).

**TABLE 1:** Five domains of situated expectancy-value theory (SEVT).

Five domains of situated expectancy-value theory (SEVT)				
Expectancy *Personal beliefs about how well the individual is likely to do*	Value *Task related beliefs about the value of anticipated outcomes and associated costs*			
Expectancy (expectancy of success)	Attainment value (importance of task)	Intrinsic value (enjoyment/interest)	Utility value (usefulness or relevance)	Associated costs (loss of time, stress)
Low/high expectation that teachers are capable of teaching remote learners using a robot for learning	Low/high value placed on personal growth and development to adapt teacher roles that meet needs of all learners	Low/high value placed on the enjoyment one expects to gain by introducing and mastering the use of robots in the classroom	Belief that mobile robots are a valuable digital and physical tool for teaching remote students	Perceived costs that might be incurred in adapting teacher roles for inclusion of mobile robots in learning experiences

### 2.3 Telepresence robots

#### 2.3.1 Commercially available telepresence robots

**FIGURE 2:**
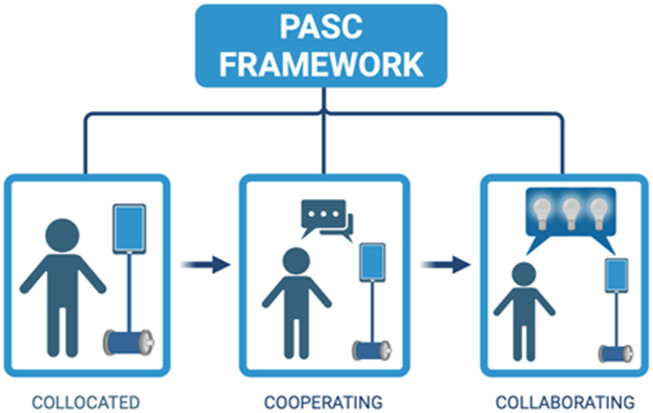
Presence and social connectedness framework.

The telepresence robot is an innovative technology that can remove the barrier of physical segregation. However, an embodied telepresence robot can provide levels of presence that vary from simply being collocated (co-present) to cooperating (following instruction) to collaborating (being richly engaged in the organic environment) as detailed in Ahumada and Eccles’ Presence and Social Connectedness framework (Ahumada-Newhart and Eccles, 2020) (Figure 2). Telepresence robots are mobile robot units that can be moved and controlled by a remote learner in a local environment (e.g., physical classroom). These robots provide real-time audio and video exchange, with the person’s face typically shown on the robot’s “head” via face screen. The remote user is in control of the movement and behavior of the robot in the local environment. This control provides the remote user a degree of embodiment in the robot and the opportunity to be present and engage in the local environment. Currently available telepresence robots differ from each other in significant ways. They have different mobility features; they may or may not allow pan and tilt of the camera; they have different microphone and speaker placements; and they have different net-work security features, among other things.

In our work, all mobile telepresence robots have the following features: Remote-controlled mobility to navigate the physical environment from a remote location. Life-size face screen that displays the remote user’s face, head, and shoulders to effectively communicate facial expressions, body gestures, and hand movements (as needed) to engage with interactants. Synchronous video and audio capabilities are also requirements of mobile telepresence robots. Recently, new additions of “robot” models have been added to the category of “telepresence robots” however, not all are mobile or have a face screen. As high value has been placed by remote users on the ability to move (walk), view (see), hear and speak (talk), desktop or non-face screened units are not covered in this study (Newhart et al., 2016; Ahumada-Newhart and Olson, 2019; Ahumada-Newhart and Olson, 2017; Ahumada-Newhart and Eccles, 2020; Ahumada-Newhart et al., 2023).

### 2.4 Summary

While prior work on Artificial Intelligence (AI) social robot systems is helpful for understanding contexts for robots in learning, these systems are designed, built, and frequently controlled by adults (Bryson and Winfield, 2017; Awad et al., 2018). Consequently, teachers using AI social robots in the classroom have different positionality and interaction scenarios with these robots than they do with telepresence robots that are actively controlled by remote learners. In this study, we explore educator perceptions of teacher roles during in-class learning activities, in-school social activities, and learning preparation for robot-mediated learning experiences. To our knowledge, this study is the first to explore educator perceptions of teacher roles in the K-12 robot-mediated learning experience.

## 3 Related theory

### 3.1 Situated expectancy-value theory

As robot-mediated learning is characterized by features of expected human-robot interaction (HRI) tasks, our study yields empirical evidence for evaluation of perceived teacher roles in the robot-mediated learning experience. Situated Expectancy-Value Theory (EVT) is a motivational theory that posits achievement-related choices are determined by two factors: 1) expectancies for success and 2) subjective task values (Eccles and Wigfield, 2002; Wigfield and Eccles, 2000; Eccles and Wigfield, 1995). That is, motivation will be highest when there is both a high expectancy of success and a high value is attached to the task. SEVT informed earlier work on a Presence and Social Connectedness (PASC) framework that was developed to gauge presence and engagement of remote students using robots in traditional classrooms (Ahumada-Newhart and Eccles, 2020). The PASC framework provides a useful heuristic for teachers to evaluate the degree to which remote learners are engaging in the learning experience with a scale from collocated to cooperating to collaborating. SEVT also assumes that teachers’ current expectancies for success and the subjective task value of various pedagogical tools will be based on prior experiences and habits. Taking on a new technique such as the use of robots in the classroom to involve remote students in classroom activities will likely pose very real challenges for teachers with limited familiarity interacting with robotic and AI technologies. To the extent that this is true, designing the robots and the technologies for using the robots needs to take into account which characteristics will lead teachers to have the highest confidence in their ability to use this tool and to attach the greatest value possible to the advantages of incorporating this technology in their classroom coupled with the lowest possible costs for such a change in their classroom dynamics.

## 4 Methodology

We used qualitative methods to explore the perspective and meaning of salient experiences, identify social structures, and identify processes in order to understand the meaning behind participant behavior (Mays and Pope, 2000; Pope and Mays, 1995).

This study employed a case study research methodology that consisted of individual interviews, group interviews, and field notes. To provide an in-depth, multidimensional study of real-world experiences of teacher roles in robot-mediated learning, data were collected from multiple sources and sites to bring out details from the viewpoints of all participants (Yin, 1994). Novelty effects were considered minimal as all participants taught (or had taught) a remote student using a telepresence robot for long-term, daily school instruction. This paper explores teacher roles, challenges, and perceptions through within-case and cross-case analyses of participants in robot-mediated learning experiences. Each case consists of an in-person teacher who taught a hybrid classroom with a remote learner who used a telepresence robot to attend class.

As a national study with remote learners from multiple states, our research was approved by university Institutional Review Board (IRB), as well as the respective IRBs and external research approval boards of our public school district partners in other states.

### 4.1 Participants

For this paper, sources of data consisted of individual and group interviews with teachers. We conducted 46 semi-structured interviews and 6 focus group interviews (n = 2; n = 3; n = 2; n = 3; n = 2; n = 2) with K-12 teachers who taught a hybrid classroom with a remote learner who used a robot to attend class. In total, the participant sample size for this study was (N60).

### 4.2 Robots used in this study

**FIGURE 3:**
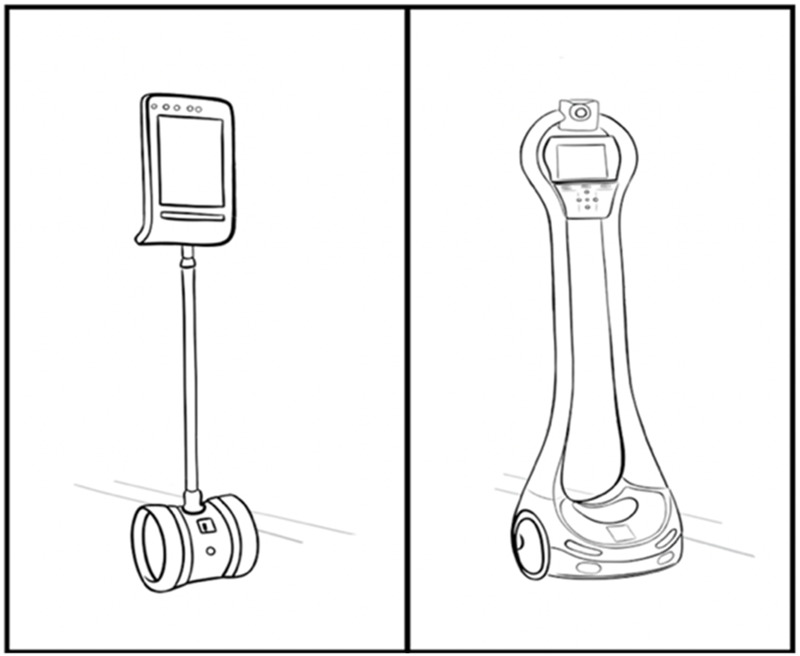
Double and VGo robots.

The robot models used in this study were the commercially available Double 2, Double 3 and VGo (Figure 3). Both models of robot offer remote controlled mobility, real-time audio/video, obstacle avoidance, and occupancy awareness. The Double robot models also offer remote controlled adjustable height for sitting and standing activities.

### 4.3 Participant recruitment and informed consent

All participants were provided with study information sheets approved by our institutional review board and local school district research offices of external research. Study information sheets were read aloud by the interviewer before each individual or group interview to provide ample time for questions about the study. Participants gave written consent before interviews were conducted. Researchers did not collect demographic data on participants, per school district guidelines.

### 4.4 Data sources

To increase trustworthiness in the data and confirm validity of the processes, we followed Yin (1994) recommendation to use multiple sources of data. Triangulation, protocols that are used to ensure accuracy and alternative explanations of the data (Stake, 2010), was accomplished by collecting data using different methods (i.e., semi-structured interviews, focus groups, observations, field notes). It was expected that the concepts and themes related to perceived teacher roles would emerge from the multiple sources of data through inductive content analysis, open coding, and the constant comparative method recommended by Glaser and Strauss (Glaser and Strauss, 1967).

Two interview methods were used in this study: focus group interviews and semi-structured interviews. Our focus group interview participants consisted of teachers who taught a hybrid classroom with a remote learner who used a robot to attend class. Focus group interviews were also conducted when more than one teacher was available at the same time at the same school. Observations and field notes were also recorded while the robots were deployed in the classroom to gain insights into participant perspectives and practices. All interviews were audio recorded, transcribed, and coded to identify patterns, similarities, and dissimilarities across all cases where each case represented one teacher.

Focus group interviews lasted 10–20 min (per district guidelines) and semi-structured interviews lasted 10–30 min. Questions covered a range of topics, including teaching experiences, adaptations to traditional teaching practices, and perceived impact on teacher workload.

### 4.5 Analysis

The process of analyzing the data involved coding and categorizing the data. We hypothesize that teachers, who choose to engage in robot-mediated learning, attach high value to inclusive education and have high confidence in robot capabilities to achieve expected teaching tasks. We employed a hybrid coding approach that combined deductive and inductive methods. We began our analysis with a set of *a priori* codes (deductive) and then added new codes (inductive) as we worked through the data. *A priori* codes (i.e., codes that are developed before examining the current data) were based on traditional teacher experiences in physical classrooms and traditional mobile telepresence robot design. Four *a priori* codes were selected to represent teacher roles from traditional organic environments: Instruction, teaching essentials, classroom environment, and robot challenges. Following Patton’s model, our analysis involved making sense of the data by reducing the volume of raw information, followed by identifying significant patterns, and finally drawing meaning from the data and building a logical chain of evidence (Patton, 2002). From this analysis, five main teacher roles emerged: capacity building, assessment, instruction, mentorship, environment design.

To explore high value teaching tasks reported by participants, and allow for new and unexpected themes to emerge from the data, we employed values coding as part of the inductive coding process. Values coding involves coding that relates to the participant’s worldview (Saldaña, 2021). In our dataset, we focused on teacher interviews that reflected the values, attitudes, and salient experiences of the participants as they related to concepts of teacher roles and practices.

To improve the systematicity, communicability, and transparency of the coding process, we employed intercoder consensus through a double coding practice where each transcript was coded by two researchers and discussed by the research team (Saldaña, 2021; Cascio et al., 2019). The coding team was comprised of one postdoctoral scholar, one graduate student, and one research associate. Initial coding was performed on transcripts following Glaser and Strauss (1967) description of open coding where tentative labels are applied to sections of data and these labels are later classified under common concepts or categories as the data undergo multiple rounds of coding. A list of the code words for each transcript was compiled and compared across the individual cases. This allowed for checks to ensure that a code was used consistently throughout the transcripts. During these steps, notes were taken and recorded of emerging codes, the ideas they represented, and relationships between codes. After the initial round of open coding, the research team discussed each coded section in terms of why it had been interpreted as meaningful and what it revealed about participant perceptions of teacher roles. After discussion, the research team agreed upon a set of codes, each with a brief definition. These codes formed the initial analytic framework. The lead researcher then independently coded each of the interview transcripts using the initial framework. Notes were taken on codes or impressions which did not fit the existing analytic framework. Codes were then refined, and new codes were introduced where necessary. The themes and concepts that emerged from the analysis were repeatedly compared with the transcripts to ensure their validity. The constant revision of the material allowed for some codes to be subsumed under broader and more abstract categories. The final code categories can be seen in Table 2.

**TABLE 2:** Codebook sample.

Codes	Definitions	Examples
Capacity building	Preparing physical instructional materials, securing digital tools, connections, access	Lesson plans, Google docs, EdPuzzles, charging robot, ensuring back-up communication channels through texts/chats
Environment design	Placement of learners, robot, desks, classroom resources	Tape on the floor for all desks so the robot had clear paths
Instruction	a) lecture-based b) discussion c) team leader	a) whole class w/robot-mediated communications b) monitor individual and one-on-one robot communications, whole class hybrid group interaction c) groupwork of >2 children, groupwork of <2
Mentorship and guidance	Individual in-class and non-robot external communications	Google docs, chats, texts, online learning platforms
Assessment and evaluation	Physical and online grading	Tests, assignments, verbal assessments

## 5 Results

**FIGURE 4:**
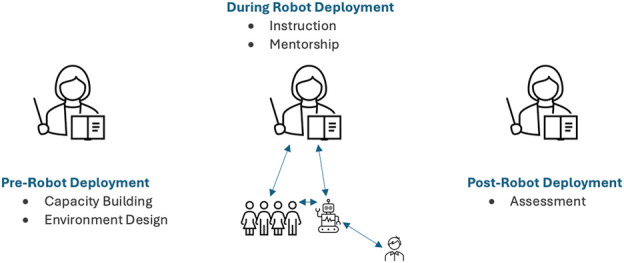
Teacher roles in robot-mediated learning.

In analyzing the data, two roles emerged as occurring before live robot deployment: capacity building, environment design; two roles emerged as occurring during live robot deployment: instruction, mentorship; and one role emerged as occurring post robot deployment: assessment (Figure 4).

Teachers were asked what impact, if any, the robot had on their teaching. As an exploratory study, teachers were given the opportunity to share salient experiences based on the teacher roles they perceived as being most impacted by the deployment of the robot in their classroom. Strong motivation to meet expected teaching goals and values emerged as a theme across all interviews. Expected teaching goals and values varied from meeting the needs of the remote student, the needs of the local students, and the needs of the teacher. Below, we present our findings per number of participants who reported adapting their teaching practices according to the five teacher role categories that emerged in the coding of the transcripts.

### 5.1 Capacity building

Transcripts were coded for capacity building when teachers reported teacher tasks related to preparing physical and digital instructional materials for the remote learner, securing digital tools, connections, and access to ensure usability of robot by the remote learner and peers. Perceived associated costs such as increased workload and preparatory time for robot-mediated learning were included in capacity building. Twenty-one teachers shared their perceived preparatory workload after a robot was deployed in the classroom (Table 3). Fourteen (67%) of these teachers felt that prep work for the remote student increased their workload, six (29%) perceived no change in their workload, and one teacher reported a perceived reduction in the workload.

**TABLE 3:** Associated costs.

Associated costs: Perceptions of preparatory workload (*n* = 21)		
Robot increases preparatory work (*n* = 14)	Robot has same preparatory work as regular student (*n* = 6)	Robot decreases preparatory work (*n* = 1)
*It’s different. There’s a lot more prep work ahead of me. I have to make sure that I have everything scanned. So, for the prep and that part, there’s that extra little bit … it’s making sure that she has everything she needs. I would say that’s the biggest challenge* *Okay, the robots coming to class. We can't sort of also have the robot and then also be sending work and then also have a home teacher . It kind of has to be one modality. Sometimes I think that can be a little challenging*	*There was this understanding that I wasn't the kind of adult that was going to come back at her and be like well why didn't you do this, when I can barely remember to do it for her. So, um yeah, we’re both very easygoing…*	*It makes may work load less, right? Because if I had to wait 6 weeks for her to come back, and for her to catch up…*

### 5.2 Environment design

Twenty-three (77%) teachers reported preparing the classroom environment for the robot. Tasks included rotating desks (to increase peer interaction with robot-mediated learner), setting up groupwork stations in hallways (for improved robot capabilities in hearing and movement), placement of the robot in the front of the classroom for improved views of the teacher and materials. Twenty-three teachers (77%) reported changing their classroom environments and eleven (37%) reported changing school environments outside the classroom to accommodate the robot during groupwork. One teacher reported restrictions on her physical movement due to the robot in the classroom (Table 4).

**TABLE 4:** Environment design.

Environment design (*n* = 30)		
Classroom environment (*n* = 23)	School environment (*n* = 11)	Restrictions on teacher physical space or robot placement (*n* = 2)
*… I rotate my seats every 2 weeks so pretty much everybody’s worked with her at some point* *I had a place in the front of the classroom where he could park and watch me when I was giving instruction, but then turn around and face the class for when he needed to interact with them* *It’s because when they move their desks and they’re not on the lines … and their desks and their chairs are crooked, that’s why he runs into them*	*if you’re doing group work and the audio sometimes picks up too much and it can't distinguish just that group and so I’ll send the kids out in the hall to work*	*You know it did make me kind of stay in one area because I didn't want her to have to be moving anything around too much. Which is hard for me because I like to move around, so it did kind of keep me stationary in one spot* *I said go to the back. I think it makes sense because you’re this big bot and you don't want to get in anybody else’s way* *The robot sat towards the middle/back, there’s not much room in the front and I need to move*

**FIGURE 5:**
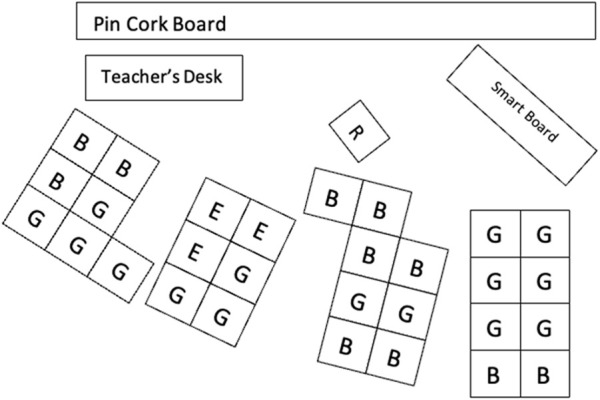
Classroom environment.

One teacher reported frustration that she could not change her classroom environment to better accommodate the robot due to a high number of students in the classroom. Figure 5 displays the placement of student desks in the classroom from the observation field notes.

*it’s very difficult to move around in the room with the robot, from a steering standpoint, and I have too many desks because I have too many kids*.

### 5.3 Instruction

Fifty-four teachers reported impacts on their instruction within three different roles—lecturer (whole class instruction with ongoing robot-mediated communications), discussion leader (monitoring learner and one-on-one robot communications during individual classwork), teams leader (monitoring learners when engaged in small groups or pairs). One teacher reported noises from the home causing disruptions. For the lecturer role, impacts were grouped into four categories that covered when a teacher had to interrupt lecture-based instruction to fix and/or aid the robot in four categories: vision, hearing/sound, mobility, no problems. Some teachers reported more impact on their instruction time (Table 5).

**TABLE 5:** Instruction--lecturer.

Instruction-Lecturer (*n* = 54)			
Vision (*n* = 20)	Hearing (*n* = 10)	Physical mobility/Movement (*n* = 5)	No issues/Robot as expected (*n* = 19)
*We’ve had the, the problems we have with the visual is the glare…The whiteboard was a little bit easier to see than the smart board* *Usually I have to pull the whole robot apart to get iPad out so I can actually manipulate the iPad around so she can see the actual lab* *It was difficult to keep my eye on the robot and the class*	*They couldn't hear because it was loud in the classroom…So then we pull up a Bluetooth speaker, but then there was connectivity issues. So they ended up just texting him* *Background noise at the house was an issue…just telling parents that she needs to be in a quiet space*	*Have it be supervised to get from class to class* *AT program for those things. And our job is to be right there to help with it*	

Twenty-two teachers reported impacts on their role as discussion leader (Table 6). Discussion leader roles occurred when learners were working on individual assignments, presentations, debates, or science labs and the teacher was moving around the room and monitoring individual learning. Perceived impacts were grouped into three categories: facilitating robot communication with the class, robot as helpful for content learning and presentations/debates, no issues reported (robot needs perceived as no different from in-person student). Some teachers reported impacts on more than one area.

**TABLE 6:** Instruction--discussion leader.

Instruction-Discussion leader (*n* = 22)		
Facilitating (*n* = 6)	Helpful (*n* = 11)	No issues (*n* = 7)
*And then I’d say okay everybody hold up, they want to say something and then everybody would turn and look at the robot and they would say what they wanted to say* *And I’ve increased my wait time [for responses]*	*The one bonus I will say about it is that he hears all my little side comments about stuff or like real world examples* *He’s seeing the kids going up and he’s seeing them like correcting sentences and stuff like that for grammar* *It still meant that he’ll be able to engage in the content, which is really the purpose when they were talking about the novels and stuff like that, she could hear what they were saying and she could talk if she wanted to in French, she would – she could hear – this is French 1*	

Thirty-seven of these teachers reported impacts on their role as teams leader (Table 7).

**TABLE 7:** Instruction--teams leader.

Instruction-Teams leader (*n* = 37)		
Facilitating (*n* = 12)	Helpful, no issues (*n* = 22)	Challenging (*n* = 3)
*A lot of times … would just carry her over to the groups and then she was just. It was like her having a chair at the back table and she would participate in group* *So I have a little room off to the side, and so if they’re working in small groups, I’ll put them in a separate room* *So, if I have her as part of a little group, I really have to plan that the other kids are independent and I’m there so I can help her communicate with her group*	*His group work in the class via the bot would be him explaining because the kids will tell him what materials they have on the desk or on the table and he will, they will ask him how he wants to either arrange it or build it, and he’ll verbally tell them and then they’ll do the steps that he’s saying. Kind of stationary, small group discussions, it worked fine* *They do a lot of small groups, so we are working on belonging problems like this. We currently use a microphone that they pass around the table*	*Say the group work is a little harder. Yes. For what you said, it’s, it’s harder for them to hear. Um, I thought, gosh, group work’s gonna be so great because that’s what they’re missing. That’s been a little more challenging. Um, so sometimes they’ll have them go in the hallway* *So it’s not I think just that, but I think with that combined with all the kids in the class talking in their own little groups makes it hard to hear* *“Okay, we’re really going to use this as it was designed,” it didn't happen. And so, the three other kids in the group did the whole project without her*

Comments on overall impacts on instruction included references to general troubleshooting, perceived workload, perceived impact of digital literacy on ease of using the robot, and associated costs in terms of lost instruction time.

*usually the negative just comes in through the, you know* “*I can*'*t get this to work.*” *Or, “It's not letting me log in.” Or, “The camera’s not working today…*“*Can you readjust it or whatever?*” *it can be a lot of work for the person who’s…hosting it* *I think some of the teachers … using Google classrooms and…very technology-based, … had an easier time* *the technical difficulties…with us at least,…detracted a little bit from the class time because we’re trying to fix her hearing or her seeing or whatever … That would take away … in terms of teaching the class.*

### 5.4 Mentorship and guidance

Mentor and guidance roles included external communications (outside the robot) with the remote learner to facilitate learning (Table 8). Most teachers used these external capabilities to provide additional instruction or guidance on class materials.

**TABLE 8:** Mentorship and guidance.

Mentorship and guidance (*n* = 33)			
Email (*n* = 7)	Cell phone/text (*n* = 10)	Google classroom (*n* = 10)	School office (*n* = 7)
*She’ll just send an email and then the one glitch with that is I’m not always looking at my email…because I’m teaching…*	*I’m just not the kind of teacher that is just going to be like, oh, I don't want them to have my cell phone number. I mean, no, I’m not giving out my number, but in this sense logistically, it was going to make my life easier for us to be able to get in touch that way* *I personally don't like giving my personal number. That’s just not something I’m comfortable with . Just giving out my cell phone number* *She has my cell phone number but I have zero [service]. Never can get texts and stuff in here*	*I would send emails and through Google Classroom. I would just send an email and let her know…*“*Here’s what we’re doing.*” *I would attach the completed copy of what we’re doing. Then, the blank copy to fill in along. And that’s how I do it. I do email and Google Classroom*	*If things didn't work, then I would talk to whoever the liaison was* [*offic*e] and say, “*Okay, well just have her work on this.*” *That was really it*

### 5.5 Assessment (*n* = 28)

Assignments delivered via sibling, home teacher, neighbor or proctor (Table 9).

**TABLE 9:** Assessment.

Assessment (*n* = 32)			
Physical materials delivered (*n* = 15)	Assessments online or on paper, real-time (*n* = 3)	Digital materials, online/Google classroom (*n* = 11)	Proctored tests/quizzes (*n* = 3)
*Access that real-time classroom stuff that the rest of the kids are having* *There’s a lot of trust that goes on when she does her graded assignments, that she’s doing them with nothing else in front of her. And there’s a lot of trust that, when she logs off before she emails it to me, that it is truly just her stuff that I’m getting. So it is a little bit more difficult*	*Did a quizzes activity, which is a web-based activity for them and she could do it right at home* *I actually have to give the hard copy to, I gave it to my assistant who’s going to send it to whoever’s at the home hospital office to get it to her* *I don't know if proctor’s the right word, but he gives them the test*	*If we were taking a test that day, then, yeah, she would log on, on her computer at home, on Google Classroom, and she would take the test just like we were doing that day*	

## 6 Discussion

Our study demonstrates five different roles teachers adapted to include remote learners via robot in the classroom. The effects of robot use on the teacher roles was significant in several settings pre, during, and post robot deployment. As most teachers reported adapting their teacher roles for instruction during deployment, three categories emerged to relay tasks associated with providing instruction to hybrid classrooms with in-person learners and remote learners using robots for learning. Additionally, strong motivation to meet expected teaching goals and values emerged as a theme across all interviews. As such, we focus our discussion on the advantages we found for using the SEVT model to understand the teachers’ reactions to having the robot in their classrooms. We also discuss the ways in which our findings can be used to improve the design of the robots in order to increase the value teachers could place on incorporating them into their classrooms.

### 6.1 During deployment—instruction

The dominant form of pedagogy used by the teachers we interviewed centered on lecture-based instruction as this style of teaching is relatively simple and reduces much of the technical and economical complexities of teaching in-person (Shulman, 2000). In robot-mediated learning, the technical and personal associated costs associated with lecturing were perceived as higher than in-person learning. The link of these teachers’ comments to the various aspects of the SEVT model were prevalent throughout the interviews. Table 10 provides a summary of several of these links of expectancy and value constructs the teachers’ instructor role during lectures, discussion, and groupwork.

**TABLE 10:** SEVT model sample, instruction-lecture.

SEVT: Instruction-lecture				
Expectancy *Personal beliefs about how well the individual is likely to do*	Value *Task related beliefs about the value of anticipated outcomes and associated costs*			
Expectancy Teachers expected the robot to work as promised, agreed to deployment in the classroom	Attainment Value High value placed on meeting needs of all learners, teachers actively engage in troubleshooting	Intrinsic Value (enjoyment/interest) Low value placed on interest in learning how to troubleshoot robots, two teachers stopped using the robots	Utility Value Persistence due to perceived usefulness of robot for learning	Associated Costs Reported loss of time, stress

Although there were examples of positive aspects of the robots during instruction, many more comments focused on the costs teachers experienced in this role. These challenges and workload occurred during lectures due to the teacher’s responsibility for technical issues with the robot. These frustrations may have occurred due to the high attainment value of lecture-based instruction for teachers because they are so focused on meeting needs of all learners through the lecture modality. If incorporating the robot in class interferes with the flow of instruction, the teachers are very likely to be concerned about the high cost of the robots in terms of the attainment value they usually experience while lecturing to their students.

The teachers’ sense of the intrinsic value of adapting to the robot may have also been low for these teachers as troubleshooting the technical complexities of a robotic unit may not have been enjoyable as repairing/and modifying technical issues is traditionally outside the scope of teacher roles. Future studies need to explore if teachers persisted in troubleshooting and repairs due to the belief that mobile robots are a valuable digital and physical tool for teaching their remote students. Additional work is also needed on the ways to improve the design of the robot-at home interface so that these costs can be reduced.

In contrast, for both the discussion and teams leader role, use of the robot was seen (at times) as helpful because the teacher’s role was centered on one-on-one assistance and guidance for learners while they completed individual and group assignments. Peers helping each other through groupwork or one-on-one conversations were also viewed as helpful to the teacher role as it involved leadership and more “troubleshooting” of challenges related to learners versus the robotic unit (Table 11). Again, having the robot in the classroom was seen as helpful in managing peer interactions that involved the child at home.

**TABLE 11:** SEVT model sample, instruction-discussions and groupwork.

SEVT: Instruction-discussions and groupwork				
Expectancy *Personal beliefs about how well the individual is likely to do*	Value *Task related beliefs about the value of anticipated outcomes and associated costs*			
Expectancy Teachers expected the robot to work as promised, agreed to deployment in the classroom	Attainment Value High value placed on meeting needs of all learners, leadership in guiding individuals and groups on learning tasks similar to in-person learners. Troubleshooting the robot may have been viewed as occupying teacher time similar to a learner requiring assistance to learn	Intrinsic Value (enjoyment/interest) High value placed on interest in learning how to assist learners and troubleshoot human learning challenges	Utility Value Perceived usefulness of robot may have been high as robot was able to receive assistance from peers and not solely reliant on teacher	Associated Costs Assisting the robot was not reported as loss of teacher time but reported as challenges faced by peer groups

#### 6.1.1 Design recommendations

Thus, even though there was evidence of robot assisted instruction facilitating teamwork, findings identify more examples of the ways in which the perceived costs of incorporating the robot into the classroom undermines both the teachers’ confidence in managing the robot and the subjective task value they placed on having the robot in the classroom. Future robot design should include reliability of robotic units in connectivity, vision, hearing, and speaking capabilities comparable to in-person learners. These features should not require teacher assistance during lectures, discussions, or groupwork. Additionally, tools for ease of communication in loud classrooms, groupwork, and one-on-one conversations should be incorporated in any robotic units deployed for learning.

#### 6.1.2 Best practices

Teachers should be provided basic “troubleshooting tips” and ample training for robot deployments. Additionally, while many teachers reported the robot requiring “buddies” to move through the school, trainings may include peer assistants who may troubleshoot quickly during instruction time. Trainings should focus on reducing the associated cost of lost instruction time related to technical aspects of the robot. As most teachers seemed aware of this associated cost both for themselves and the other learners, districts should provide increased technology services for teachers who use the robots.

### 6.2 Pre-deployment-capacity building and environment design

#### 6.2.1 Capacity building

Teacher workload managing the robot in the classroom was also reported as higher than workload for in-person students. Some of the identified environmental challenges involved crowded classrooms and dynamic environments where peers and janitorial staff moved desks that were carefully aligned to facilitate robot mobility. Preparing the physical classroom, finding appropriate placement for the robot, and balancing a teacher’s value on moving during instruction versus the robot’s need to be in front of the room were also reported as challenges. In one case, high value was placed on the robot being able to see instructional materials on the board even though this caused the teacher to restrict her own movements during instruction. In another case, high value was placed on the teacher’s need to move and express herself in the front of the room with placement of the robot in the middle/back of the room so it would not get in her way or obstruct the views of other students. Understanding teacher and peer expectancy and values provides support for the capacity building tasks necessary prior to placement of the robot.

#### 6.2.2 Design recommendations

Making the robots work in classrooms clearly requires greater flexibility in the placement of desks to allow mobility of the robot, just as would be required to adapt to learners who uses a walker or wheelchair.

#### 6.2.3 Best practices

Awareness of existing district supports and how they translate to robot-mediated learning. Districts should provide increased technology support and reduced class sizes when robots are deployed.

#### 6.2.4 Post-deployment - assessment

Teachers also reported assessment challenges with the amount of organization and workload required to provide remote students with materials, tests, and quizzes. As different districts provide different resources, teachers were left to adapt their teacher roles to robot use based on current practices. Not all practices were useable for robot-mediated learning. Teachers with home instruction services sent assignments home with the home teacher, those who were aware of social support systems sent assignments home with siblings and neighbors. Teachers who were already using online platforms, such as Google Classroom, relied on the platform for delivery of assignments, tests, and quizzes. However, challenges were perceived with inequitable “honor systems” and the need for proctors.

#### 6.2.5 Design recommendations

To date, commercially available robots do not have capabilities for writing on paper and completing assessments in the classroom alongside in-person learners. Future robots may be designed to include software tools that coordinate educational online platforms with in-class assessments. Robot user interface software may be coordinated with physical robot displays as learners complete assessments in real-time while being monitored by the teacher or an aide (similar to in-person assessments). As many teachers reported using online educational platforms for assessments, shared screen capabilities may also reduce the need for home delivery, honor systems, and/or proctors.

#### 6.2.6 Best practices

Similar to capacity building, awareness of existing district supports and how they translate to robot-mediated learning should be included in professional development. Districts should provide increased technology support and reduced class sizes when robots are deployed.

## 7 Conclusion

The aim of this work was to explore educator perceptions on teacher roles in the robot mediated learning experience. Our contributions to the field include a descriptive study of educator perceptions of adaptations to teaching roles pre, during, and post robot deployments in the classroom. Additionally, we contribute a framework based on Situated Expectancy-Value Theory (SEVT) to support future studies on how teachers facilitate learning for remote learners using mobile telepresence robots. Our SEVT tool allows for mapping of individual teachers’ expectancy and values that inform how they will use the robot. This tool may increase understanding of challenges and successes in the robot-mediated learning context for teacher roles. Additionally, this tool may be used to map task values for teachers, peers, and remote learners. These task values may identify human expectations and values that inform future robot design for learning environments.

## Data Availability

The datasets presented in this article are not readily available because Sample size is too small and number of educational partners are limited, sharing of datasets may reveal identity of participants. Requests to access the datasets should be directed to vahumada@ucdavis.edu.
